# Potential Role of APEX1 During Ferroptosis

**DOI:** 10.3389/fonc.2022.798304

**Published:** 2022-03-03

**Authors:** Nan Guo, Yan Chen, Yuhong Zhang, Yonghao Deng, Fancai Zeng, Xiang Li

**Affiliations:** ^1^ Department of Biochemistry and Molecular Biology, School of Basic Medical Science, Southwest Medical University, Luzhou, China; ^2^ Department of Dermatology, The Affiliated Hospital of Southwest Medical University, Luzhou, China

**Keywords:** APEX1, ferroptosis, HIF, Nrf2, p53

## Abstract

Ferroptosis is a recently discovered category of programmed cell death. It is much different from other types of cell death such as apoptosis, necrosis and autophagy. The main pathological feature of ferroptosis is the accumulation of iron-dependent lipid peroxidation. The typical changes in the morphological features of ferroptosis include cell volume shrinkage and increased mitochondrial membrane area. The mechanisms of ferroptosis may be mainly related to lipid peroxidation accumulation, imbalance in amino acid antioxidant system, and disturbance of iron metabolism. Besides, hypoxia-inducible factor (HIF), nuclear factor-E2-related factor 2 (Nrf2), and p53 pathway have been demonstrated to be involved in ferroptosis. At present, the molecular mechanisms of ferroptosis pathway are still unmapped. In this review, an outlook has been put forward about the crucial role of apurinic/apyrimidinic endodeoxyribonuclease 1 (APEX1) in the regulation of ferroptosis. APEX1 plays an important role in the regulation of intracellular redox balance and can be used as a potential inhibitor of ferroptotic cell death. Bioinformatics analysis indicated that the mRNA level of APEX1 is decreased in cases of ferroptosis triggered by erastin. Besides, it was found that there was a significant correlation between *APEX1* and genes in the ferroptosis pathway. We have discussed the possibility to employ APEX1 inducers or inhibitors in the regulation of ferroptosis as a new strategy for the treatment of various human diseases.

## Introduction

Cell death plays an important role in the development, aging, tissue homeostasis, immunity, and stress tolerance of all multicellular organisms. There are several types of cell death, such as apoptosis, necrosis, and autophagy. These different types of cell death play an important role in the progression of various diseases, such as cancer (1), neurodegenerative diseases (2), and cardiovascular diseases (3). A variety of drugs has been used to target these types of cell death in order to treat many human diseases. Ferroptosis is a novel type of cell death discovered by Dixon in 2012 (4). The mechanisms of ferroptosis are generally accompanied by the accumulation of large amount of iron and lipid peroxide in cells. The main characteristics of ferroptosis include a wrinkling of mitochondrial morphology and loss of mitochondrial cristae, while no nuclear sequestration or chromatin border set is found. Also, in contrast to the classical autophagy pathway, ferroptosis does not form any autophagic lysosome structure (4).

Iron is an essential trace element found in all organisms and it plays a key role in the smooth functioning of the body. Cellular iron uptake is mediated mainly by transferrin (Tf) and transferrin receptor1 (TfR1) (5). Under normal physiological conditions, extracellular iron binds to Tf, and in turn Tf binds to TfR1 that is present on the surface of the cell membrane to form iron-Tf-TfR1 complex. Thus formed iron-Tf-TfR1 complex enters the cell through endocytosis. Under the action of divalent metal transporter 1 (DMT1), the extracellular iron enters the cytoplasm forming labile iron pool (LIP) that can be used by the mitochondria or it can also be used by the cytoplasm itself. This intracellular iron also can be stored by ferritin or it can be transported to the exterior of the cell through ferroportin 1 (FPN1) (6). Therefore, under pathological conditions, intracellular iron overload is an important cause of ferroptosis. Zhang et al. have reported that ferroptosis triggered by iron overload plays an important role in Parkinson’s disease (7). Besides, many studies have reported that excess free iron can generate reactive oxygen species (ROS) through Fenton reaction (8, 9). Iron circulates between the reduced and oxidized states, resulting in the formation of free radicals. There is no doubt that the occurrence of ferroptosis is directly related to the presence of iron in the cell. Hence, level of iron present in the cell directly determines the mechanism of ferroptosis.

Cystine/glutamate transporter (system XC-) is an important transporter that mediates the exchange of extracellular cystine (Cys2) and intracellular glutamate (Glu). It is composed of two subunits, namely, solute carrier family 7 member 11 (SLC7A11) and solute carrier family 3 member 2 (SLC3A2). The basic function of system XC- is to absorb Cys2 and excrete Glu. First, Cys2 is absorbed by system XC- and reduced to cysteine (Cys). Next, Cys takes part in the synthesis of glutathione (GSH) (10). Later, GSH can convert toxic lipid hydroperoxides (L-OOH) into non-toxic lipid alcohols (L-OH) by using GSH peroxidase 4 (GPX4) (11). Therefore, inhibition of GSH synthesis will lead to oxidative damage, thereby resulting in cell death. GPX4, (a member of the GPXs family), plays an important role in ferroptosis. The main function of GPX4 is promotion of decomposition of hydroperoxide and protection of the structure and function of the cell membrane from oxidative damage (12). Therefore, inhibition of GPX4 synthesis will lead to oxidative damage or ferroptosis (13). It can be understood that there is increasing evidence stating that the inducer of ferroptosis, such as erastin (14), RSL3 (15), has a promising future in the treatment of cancer. At the same time, the inhibitor of ferroptosis such as ferrostatin-1 (16), liproxstatin-1 (17), etc. can contribute to the treatment of various nervous system diseases and cardiovascular diseases.

Apurinic/Apyrimidinic endodeoxyribonuclease 1 (APEX1), also named as reduction-oxidation factor-1 (Ref1), belongs to the DNA repair enzymes. It is a multifunctional protein and plays a central role in the cellular response to oxidative stress. On the other hand, APEX1 plays an important role in the repair of oxidized and alkylated genomic DNA bases by identifying and cleaving nucleotide chains at 5’ apurinic (AP) sites (18). APEX1 also exerts reversible nuclear redox activity to regulate DNA binding affinity and transcriptional activity of transcriptional factors by controlling the redox status of their DNA-binding domain. In this review, we mainly focus on the function of APEX1 in antioxidant and regulating transcriptions. Previous studies have reported that APEX1 overexpression enhances the ability to resist oxidative stress and reduces the levels of ROS (19, 20). Besides, Daniel et al. have demonstrated that the accumulation of iron inhibits the activity of APEX1 (21). It has been well researched and concluded that the accumulation of iron and ROS is an important cause of ferroptosis (4). APEX1 is originally identified as a DNA repair enzyme and shown to be important for the base excision repair pathway (22, 23). In addition, it was demonstrated that APEX1 reduces a redox-sensitive cysteine residue of the transcription factor activator protein-1 (AP-1) and thereby facilitates its DNA-binding and transcriptional activities (24). There is increasing evidence that APEX1 is widely involved in various diseases caused by oxidative stress, such as Parkinson’s disease (20), ischemic stroke (25), Alzheimer’s disease (26), and cancer (27). Besides, many studies have proven that ferroptosis is involved in the occurrence as well as the development of these diseases. Therefore, we suggest that APEX1 may inhibit ferroptosis. However, further mechanistic details of the APEX1-ferroptosis link remain to be unclear. In this review, we highlighted the potential roles of APEX1 in ferroptosis. We hope to further explore the ferroptosis signaling pathway. It can be suggested that APEX1 is involved in various diseases through ferroptosis pathway. In the future, APEX1 will be an important target for the treatment of various human diseases.

## APEX1 and Lipid Peroxidation in the Ferroptosis Pathway

The abnormal metabolism of amino acids is closely related to ferroptosis. Mounting evidences have reported that the absence of cysteine leads to the decrease of GSH synthesis, thereby resulting in the inactivation of GPX4 and the accumulation of lipid peroxidation (28–30). There are two forms of glutathione, namely reduced glutathione (GSH) and oxidized glutathione (GSSG). The antioxidant function of GSH relies on its sulfhydryl group, which is derived from cysteine. Therefore, the levels of intracellular cysteine affect the activity of GPX4, thereby affecting ferroptosis. The accumulation of lipid peroxidation caused by the inactivation of GPX4 is one of the important reasons to induce ferroptosis. Jie et al. have reported that APEX1 overexpression inhibits the accumulation of ROS and the decrease of GSH level (31). The decrease of GSH expression affects the activity of GPX4, indirectly. Studies have demonstrated that the lipid peroxidation and APEX1 expression are significantly higher in the tumor tissue compared to the non-tumor regions (32–36). We suggest that APEX1 overexpression can inhibit the accumulation of lipid peroxidation, thereby promoting the survival of the tumor cells. Due to the function of APEX1, APEX1 may be related to the repair of DNA damage, on the other hand, APEX1 is involved in activation of several antioxidant transcription factors. Thioredoxin-1 (Trx-1) is an important part of antioxidant system in cells (37). Earlier reports have demonstrated that Trx-1 can associate directly with APEX1 in the nucleus (38, 39). Hirota et al. have suggested that AP-1 activation in response to ionizing radiation involves the passage of redox signals through Trx-1 from the cytoplasm to the nucleus, followed by interaction with APEX1 (40). Besides, Ando et al. have found that APEX1, as a redox chaperone, can regulate DNA-binding activities of various transcription factors through promoting the reduction of their critical cysteine residues by other reducing molecules such as GSH and Trx-1 (41). Trx-1 also can inhibit the accumulation of lipid peroxidation (42, 43). Recently, Trx-1 has been proven to be an inhibitor of ferroptosis (44, 45). Therefore, these results suggested that APEX1 can inhibit the accumulation of lipid peroxidation by regulating the expression of antioxidant factors through the interaction with Trx-1.

## APEX1 and System XC- in the Ferroptosis Pathway

GPX4 and system XC- are considered to be the main signaling pathways of ferroptosis (4). System XC- belongs to the family of heterodimeric amino acid transporters. The main function of system XC- is to exchange cystine with glutamate (46). It is well known that cisplatin is a very effective and widely used anticancer drug (47). Earlier reports have reported that the mechanism of action of cisplatin is mainly through the production of ROS, leading to apoptosis in osteosarcoma (48). Recent studies have suggested that cisplatin is also an inducer of ferroptosis (49–51). Tetsuro Sasada et al. have discovered the cisplatin-resistant variants of HeLa cells and the cisplatin-resistant cells also showed enhanced cystine uptake, which led to a significant increase in the content of intracellular sulfhydryl as well as GSH (52). Interestingly, overexpression of APEX1 can inhibit the accumulation of ROS induced by cisplatin (48, 53). Therefore, these findings suggested that APEX1 may play a potential role in the inhibition of cisplatin-induced ferroptosis through scavenging ROS. Also, APEX1 may be a key target in the ferroptosis pathway.

## APEX1 and Nrf2 in the Ferroptosis Pathway

**Figure 1:**
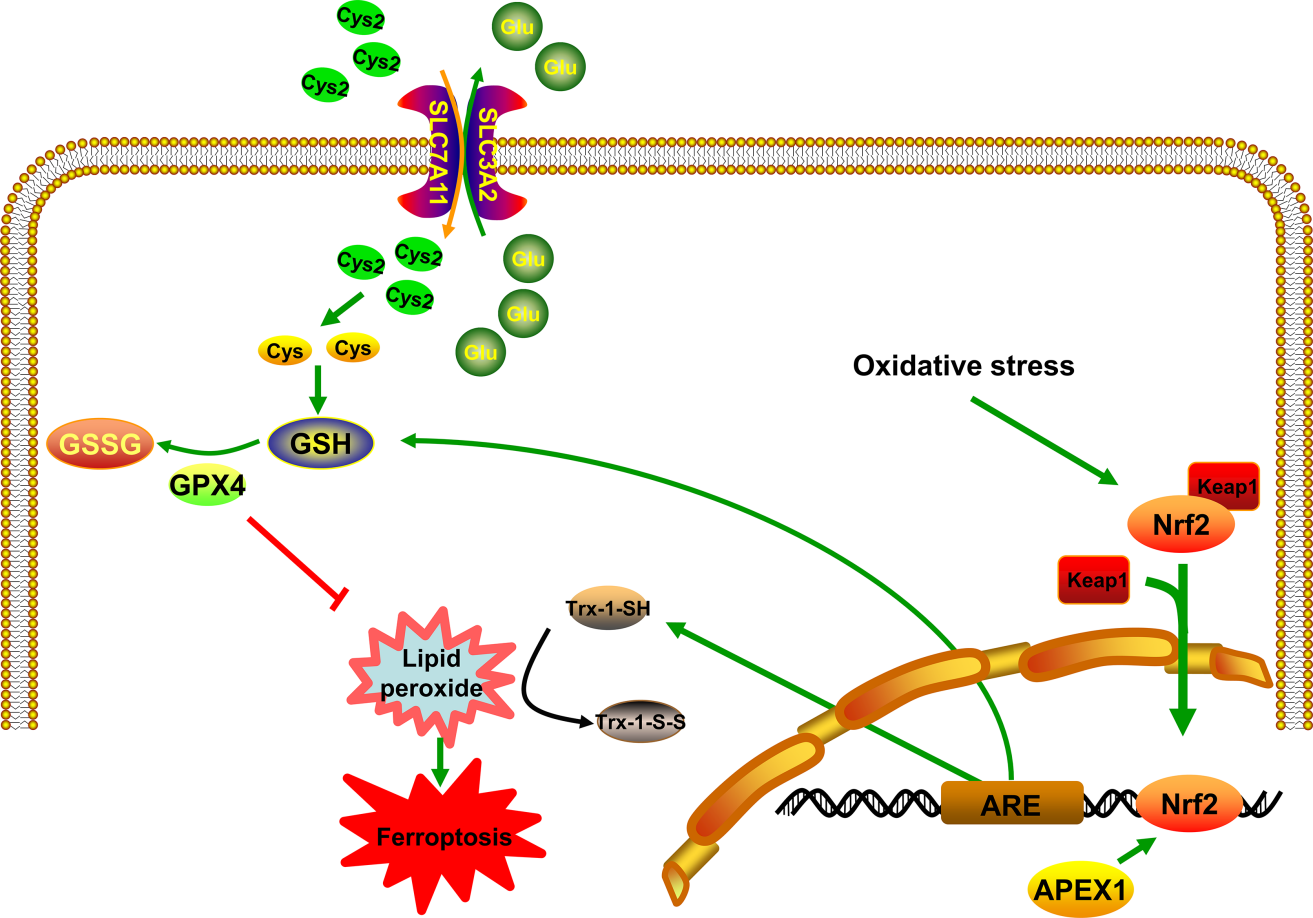
APEX1 inhibits ferroptosis by regulating Nrf2/ARE pathway. APEX1 induces the expression of ARE by regulating the activity of Nrf2. Trx-1 and GSH resist the accumulation of lipid peroxidation through its sulfhydryl group.

Nuclear factor-E2-related factor 2 (Nrf2) belongs to a small family of transcription factors, which has the oxidation resistance function. Kelch-like ECH-associated protein 1 (Keap1) binds to Nrf2 and causes rapid ubiquitination and degradation. It then inhibits the transcriptional activity of Nrf2. Under stressful conditions, the cysteine residues of Keap1 get modified, causing it to lose its ability to ubiquitinate Nrf2. This causes Nrf2 to enter the nucleus and activate a series of downstream target genes (54) (**Figure 1**). Recently, many studies have proven that the Nrf2/Keap1 pathway is closely related to ferroptosis. Fan et al. have suggested that activation of the Nrf2 pathway can upregulate the system XC- and overexpression of Nrf2 or knockdown of Keap1 expression promotes resistance to ferroptosis (55). Besides, there is increasing evidence suggesting that inhibition of the Nrf2/Keap1 pathway by targeting GPX4 could reverse the resistance towards ferroptosis (56–58). In other words, the activation of the Nrf2 pathway can induce the expression of antioxidant genes in the nucleus, thus resisting ferroptosis. In immunoprecipitation reaction experiment, Thakur et al. have found that APEX1 and Nrf2 physically interacts, which suggests that APEX1 mediates Nrf2 activation in lung cancer cells (59). Besides, Sriramajayam et al. have demonstrated that APEX1 is required for the activation of Nrf2 and subsequently activates the expression of antioxidant responsive element (ARE). The relationship between APEX1 and Nrf2 may be involved in the redox function of APEX1, which might be directly regulating the ARE-mediated neuronal survival (60) (**Figure 1**). To put it in other words, important oxidative stress genes, including *Txnrd1*, *Hmox1*, and *Gpx4*, which are involved in ferroptosis, will be expressed. These results implied that the crucial functions of APEX1 interacting with Nrf2 activates the expression of ARE and then resists ferroptosis induced by ROS. More importantly, many studies have confirmed that the relationship between APEX1 and Nrf2 is crucial for maintaining the homeostasis of ROS (60). It is now known that the APEX1/Nrf2/Keap1 is involved in a variety of human diseases and plays a significant role in Alzheimer’s disease (61) and cancer (59). All of these diseases have been reported to be closely related to ferroptosis. Therefore, APEX1 could possibly play a crucial role in the ferroptosis pathway (**Figure 1**).

## APEX1 and p53 in the Ferroptosis Pathway

**Figure 2:**
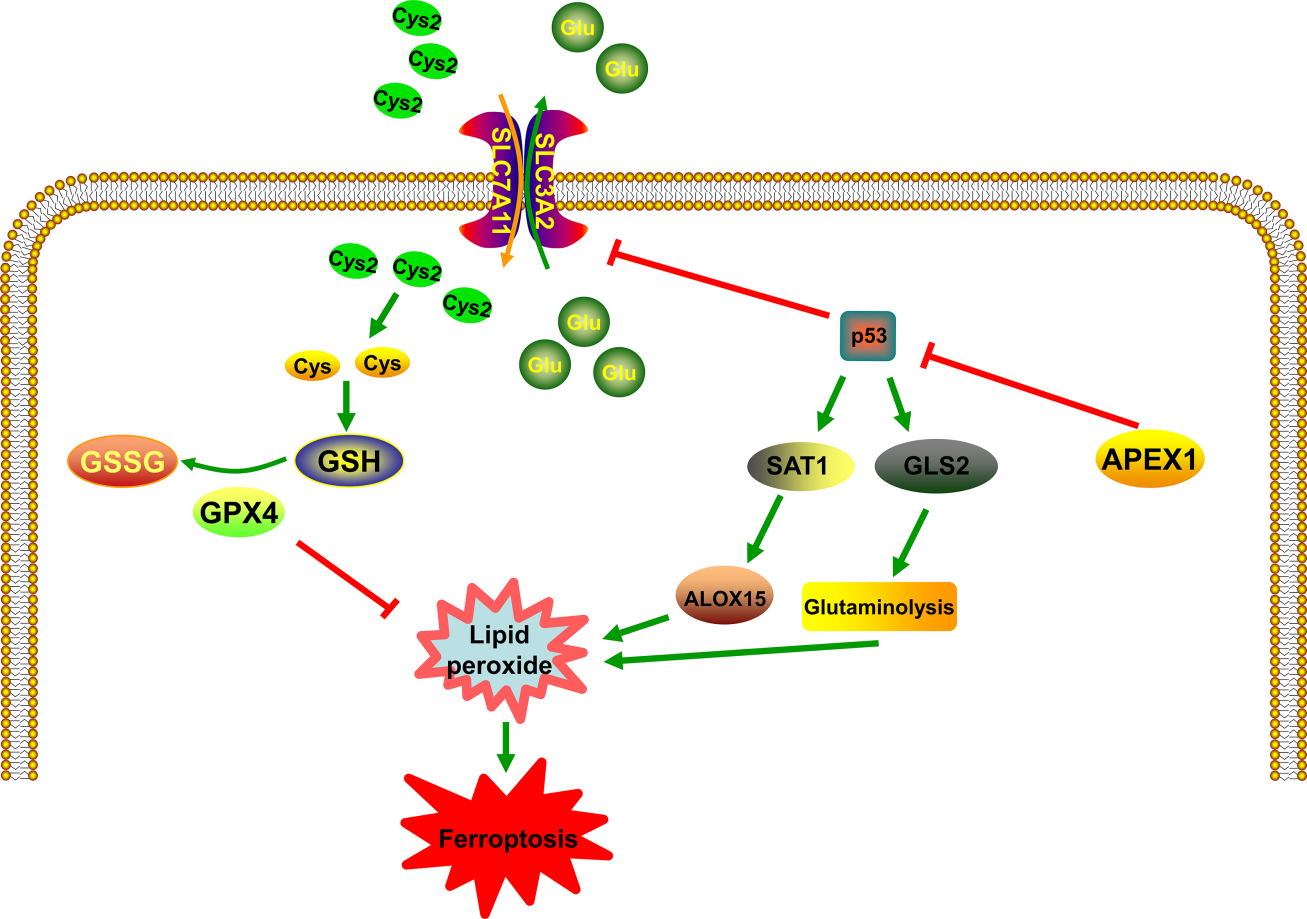
The relationship between APEX1 and p53 in the control of ferroptosis. p53 plays an important role in the regulation of lipid peroxidation in ferroptosis. p53 can promote ferroptosis through the inhibition of system XC- expression or the induction of SAT1 and GLS2 expression. APEX1 is involved in the development of ferroptosis by regulating p53.

Tumor suppressor protein p53 (TP53) plays an important role in cellular stress response to DNA damage and hypoxia (62). Low levels of stress can trigger the activation of p53, which induces cell cycle arrest, DNA repair, and survival of the cell. Downregulating intracellular ROS, p53 can protect the cells from oxidative stress-induced DNA damage and cell death. On the contrary, high levels of stress lead to the activation of p53, which induces apoptosis and cell death (62). In the recent past, p53 has been identified as a novel regulator in ferroptosis (63–65) and it has been identified to inhibit SLC7A11 gene transcription in ferroptosis (66) (**Figure 2**). Celia R. Berkers et al. have proposed that p53 inhibits the fatty acid synthesis and promotes fatty acid oxidation, thus playing a negative regulatory role in lipid synthesis (67). Ou et al. have proven that p53 can induce ferroptotic responses by directly activating its target gene *SAT1*, which is correlated with the expression level of arachidonate 15-lipoxygenase (ALOX15) (68) (**Figure 2**). Silencing of ALOX15 significantly decreases both RSL3-induced and erastin-induced ferroptosis *in vitro* (69). Activation of p53 significantly decreases system XC- expression (70). Upregulated p53 expression by Tanshinone IIA results in the destruction of cysteine import, which reduces glutathione production and promotes ROS mediated ferroptosis (71). Besides, it is known that p53 can enhance ferroptosis by promoting glutaminase 2 (GLS2). Knockdown of GLS2 expression inhibits ferroptosis through control of glutaminolysis (72) (**Figure 2**). Additionally, a large number of studies have shown that there is an important regulatory relationship between APEX1 and p53. Zhu et al. have found that Genistein stabilizes p53 through targeting interaction between APEX1 and p53. The interaction between APEX1 and p53 can promote p53 degradation. Interestingly, under oxidative stress condition, APEX1 will be oxidized by ROS and dissociate from the p53, and thus to stabilizing p53 (73). According to the above research, the possible role of APEX1 as an oxidative stress sensor may inhibit ferroptosis by interacting p53 protein. Additionally, Cun et al. have proven that downregulation of APEX1 can enhance the sensitivity of p53 mutant tumor cells to radiotherapy *in-vitro* and *in-vivo*. In other words, the possible mechanism of APEX1 regulating p53 represented that APEX1 is involved in the development of ferroptosis. Several studies have proven that APEX1 is overexpressed in many human tumors (74). In addition, tumor tissues also exhibit increased levels of ROS than normal tissues (75, 76). However, under sustained oxidative stress, tumor tissues become well-adapted to such stress through a series of mechanisms, and it often has defects in the mechanism of cell death, which is one of the primary reasons for drug resistance. Therefore, we realize that overexpression of APEX1 in tumor cells may be a protective mechanism to resist the accumulation of lipid peroxidation and make tumor cells survival. APEX1 overexpression in tumor cells could be owing to the following reasons: one is to resist the high levels of ROS; the other is to prevent the death of tumor cells by resisting apoptosis and ferroptosis by interacting p53 protein. This may be the reason for the high expression of APEX1 in tumor cells.

## APEX1 and Hypoxia-Inducible Factor-1 (HIF-1) in the Ferroptosis Pathway

**Figure 3:**
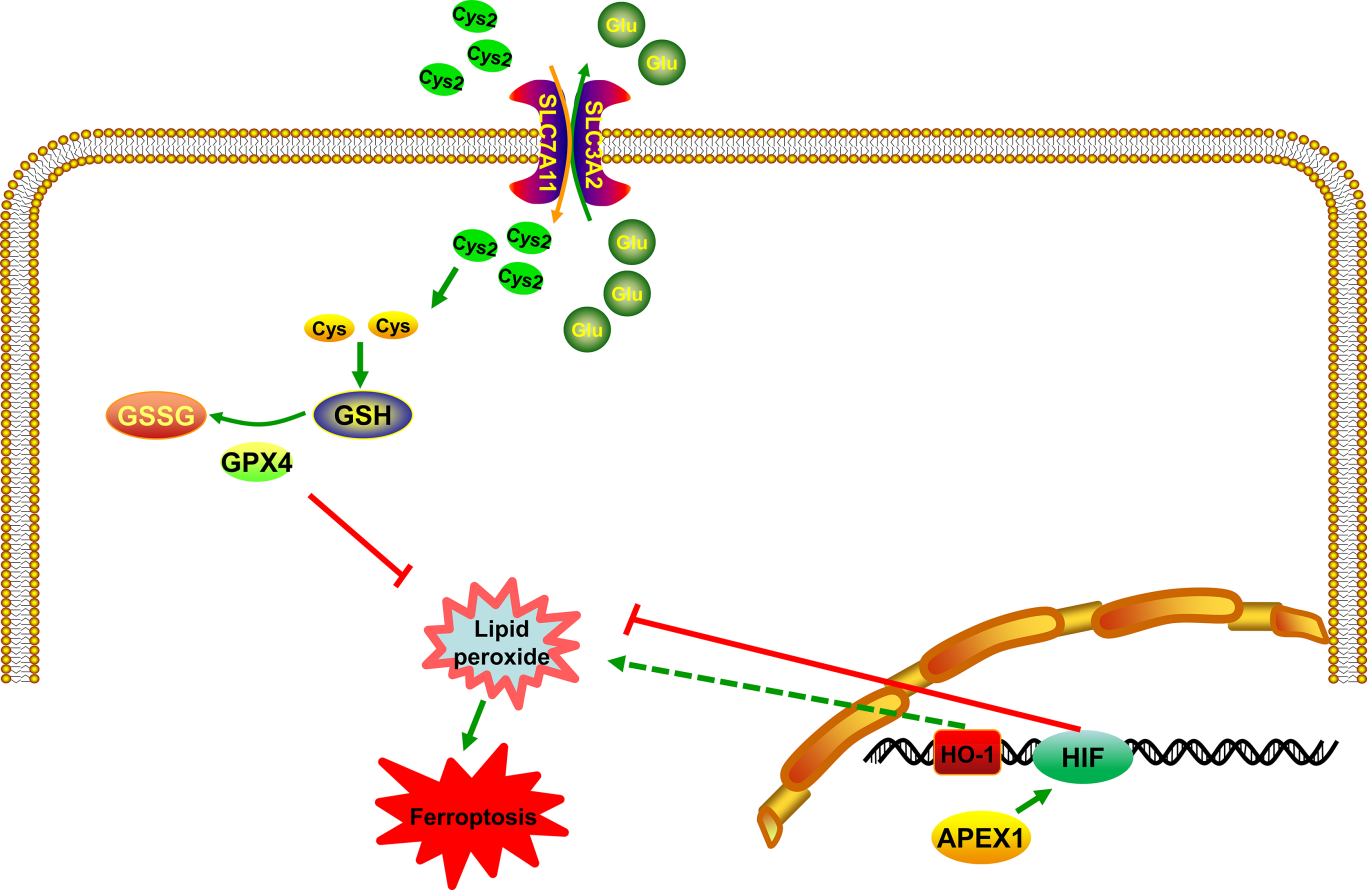
APEX1 inhibits ferroptosis by regulating the HIF pathway. APEX1 regulates regulate ferroptosis by interacting with HIF. HO-1 has a dual role in ferroptosis.

HIF-1 is a transcriptional complex that plays an important role in the regulation of gene expression by oxygen. It was first identified as a transcriptional activator, binding to the hypoxia response element (HRE) in the promoter region of erythropoietin (77). HIF consists of a constitutively present β subunit and oxygen-regulated α subunit. HIF subunits can be divided into three types: HIF-1α, HIF-2α, and HIF-3α. Among them, HIF-1α is the most widely studied α isoforms and expressed in all human cell types. Recently, studies have demonstrated that HIF-1 is involved in many diseases by regulating ferroptosis pathway, such as stroke (78), cancer (79) and some other neurological diseases (80). The rapid growth of tumor will lead to local hypoxia. HIF-1α can activate the expression of downstream target gene vascular endothelial growth factor (VEGF), induce tumor cells to generate blood vessels, and bring oxygen and nutrients to tumor cells. Inhibitors of HIF-1α can suppress the proliferation, growth, metastasis and invasion of tumor cells. More importantly, current studies have reported that HIF-1α can limit ferroptosis by influencing lipid metabolism and storing lipids in droplets (81), thus attenuating peroxidation-mediated damage (82). Moreover, recent studies have demonstrated that HIF-1α can activate the Nrf2 pathway to protect from ischemia-reperfusion cardiac (83) and skeletal muscle injuries (84). Heme oxygenase-1 (HO-1) is one of the HIF target genes (85). Lately, it has been reported that HO-1 has a dual role in ferroptosis (86, 87) (**Figure 3**). Feng et al. have reported that ferrostatin-1, a ferroptosis inhibitor, can resist ferroptosis induced by diabetic nephropathy by regulating HIF-1α/HO-1 pathway (88) (**Figure 3**). Besides, in the brain, M30, as an iron chelator, can stabilize HIF-1α (89). This leads to the ability of iron chelators to stabilize HIF-1α thereby adding to the protective effects, through their ability to prevent the accumulation of lipid peroxide *via* the Fenton reaction. Interestingly, Bianchi et al. have reported that the iron deprivation can stimulate TfR transcription by regulating HIF-1α (90). These results confirmed that HIF-1α is a key factor in ferroptosis. Stabilizing of HIF can probably inhibit the accumulation of lipid peroxide and promote the expression of VEGF, which contributes to survival of tumor. In other words, it may inhibit ferroptosis, indirectly. Latest researchers have mentioned that ferroptosis is involved in many nervous system diseases and inhibition of HIF can lead to neuronal cell death. Regulating the expression of HIF may be a potential target in ferroptosis. APEX1 can promote DNA binding activity by regulating a variety of transcription factors, including HIF (91). Huang et al. have demonstrated that overexpression of APEX1 significantly potentiates hypoxia-induced expression of a reporter construct containing the wild-type HIF-1 binding site (92). Logsdon et al. have reported that APEX1 interacts with HIF-1α under hypoxia and inhibition of APEX1 will result in decreased HIF-1α–mediated induction of carbonic anhydrase IX, which will inhibit viability of cancer cells (93). At present, no HIF-1-specific inhibitors currently exist, so targeting APEX1 regulate HIF activity is a promising method to modulate ferroptosis signaling in tumor. Interestingly, a previous *in-vitro* study has proven that an inhibitor of APEX1, E3330, lead to tumor growth inhibition (94). Besides, E3330 exposure promotes endogenous ROS formation in pancreatic cancer cells, thereby inhibiting cancer cell growth and migration (95). Based on the above research findings, it can be mentioned that APEX1 acts as an endogenous antioxidant factor in response to acute and chronic oxidative stress conditions by regulating HIF pathway. The accumulation of ROS and HIF pathway are involved in ferroptosis, however, there has been no reports that whether APEX1 plays a key role in regulating ferroptosis. These results indicated that APEX1 may be involved in ferroptosis by regulating transcription factors through its antioxidant capacity.

## Discussion

Ferroptosis is a new type of cell death found in recent years. It is involved in a variety of molecular expression and signaling pathways. The metabolic imbalance of iron and the accumulation of lipid peroxide are the primary mechanisms of ferroptosis. Since the discovery of ferroptosis, the specific molecular mechanism still remained unmapped. However, many studies have confirmed that ferroptosis pathway is involved in the occurrence and development of many human diseases such as cancer (96), kidney diseases (97), and brain diseases (98). With the development of drug research, we have also identified several highly effective drugs that can be used in the targeting of ferroptosis to treat various diseases such as acetaminophen, sulfasalazine, and so on. Potential mechanisms of drugs used in the treatment of diseases by targeting ferroptosis are shown in **Table 1**. In this review, the possible molecular mechanisms of ferroptosis signaling pathways have been focused, and it is observed that the expression and activity of APEX1 play a vital role in regulating ferroptosis pathways, including system XC-, lipid peroxidation, Nrf2, p53 and HIF. APEX1 is a dual-function protein containing a redox domain and a DNA repair domain. Besides, APEX1 also can act as an essential transcription factor to regulate gene expression (18). Okazaki et al. have proven that APEX1 functions as a trans-acting factor that binds to negative calcium response elements (nCaRE) complex in the human *PTH* gene promoter (131). In addition, recent studies have reported that APEX1 plays a key role in the regulation of apoptosis (132) and autophagy (133). In this review, we report the possible mechanisms of APEX1 regulating ferroptosis, and these mechanisms beckon further in-detail studies. At least, we indicate that APEX1 may inhibit ferroptosis by regulating redox balance through regulating transcription factors. A previous study has demonstrated that Trx-1 inhibits 1-methyl-4-phenyl-1,2,3,6-tetrahydropyridine (MPTP)-induced ferroptosis by upregulating the expression of GPX4 (45). Besides, Evijola Llabani et al. have proven that the Trx-1 inhibitor can cause the occurrence of ferroptosis, directly (44). Importantly, we highlight that there is an interaction between APEX1 and Trx-1. In other words, APEX1 may be an important molecule in the ferroptosis pathway by inhibiting the accumulation of lipid peroxidation. In addition, APEX1 may affect ferroptosis by regulating other pathways. Owing to the role of APEX1 in many signaling pathways, the inducer or inhibitor of APEX1 may be a more effective drug for the treatment of various clinical diseases. For example, APEX1 can inhibit both apoptosis and ferroptosis, so it may be more effective than other drugs in the treatment of various diseases. At present, the inhibitor of APEX1, E3330, has been used to inhibit tumor migration in pancreatic cancer and non-small cell lung cancer cells (134). Previous studies have reported that Parkinson’s disease can cause neuronal ferroptosis (135, 136). Importantly, Kang et al. have demonstrated that APEX1 overexpression inhibits the increase of ROS induced by Parkinson’s disease model (20). Therefore, APEX1 may be a promising target to treat cancer and nervous diseases by regulating ferroptosis.

**Table 1:** Potential mechanisms of drugs in the treatment of diseases by targeting ferroptosis.

Type	Name	Target	Application	Reference
**Inducer**	Erastin	System XC-	Lung cancer, Melanoma, Breast cancer, Gastric cancer, Ovarian cancer	(14, 99–102)
	Sorafenib	System XC-	Hepatocellular carcinoma	(58)
	Sulfasalazine	System XC-	Breast cancer, Head and neck cancer, Pancreatic cancer	(103–105)
	RSL3	GPX4	Colorectal cancer, Head and neck cancer, Cardiomyocytes	(56, 106, 107)
	ML-162	GPX4	Head and neck cancer	(56)
	Acetaminophen	GPX4	Lung cancer, Liver injury	(14, 108)
	Withaferin A	GPX4	Neuroblastoma	(109)
	Artesunate	Iron	Liver fibrosis, Head and neck cancer, hepatocellular carcinoma	(50, 110, 111)
	Cisplatin	GSH	Head and neck cancer, Gastric cancer, Kidney injury	(50, 112, 113)
**Inhibitor**	Deferoxamine	Iron	Spinal cord injury, Chronic obstructive pulmonary disease, Primary neurons Hypoxia, Pancreatic cancer	(114–118)
	Ferrostatin-1	Lipid peroxidation	Lung injury, Cardiomyopathy, Cell model of Parkinson’s disease, Intracerebral hemorrhage, Epilepsy	(16, 119–122)
	Liproxstatin-1	Lipid peroxidation	Morphine tolerance, Ischemia/reperfusion injury, Acute renal failure	(123–125)
	Vitamin E	Lipid peroxidation	Hepatocellular degeneration, Sepsis, cognitive impairment	(126–128)
	Baicalein	Lipid peroxidation	Traumatic brain injury, Posttraumatic Epileptic Seizures	(129, 130)

**Figure 4:**
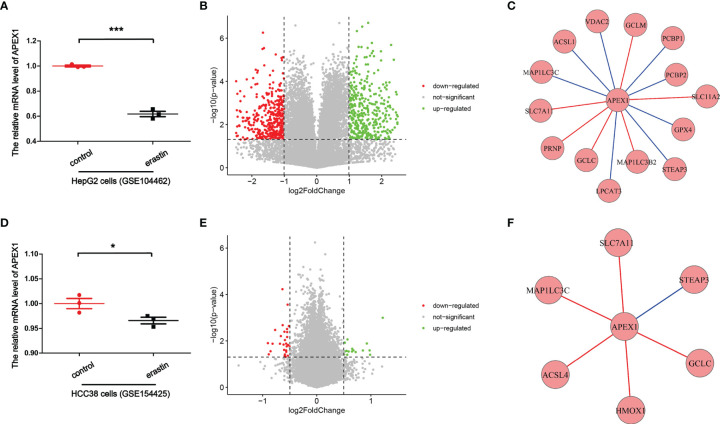
Bioinformatics analysis for *APEX1* transcriptional expression. **(A)** The relative mRNA level of APEX1 in HepG2 cells. (GSE104462) (***P < 0.001, n = 3). **(B)** Volcano plot showed significantly differentially expressed genes. (GSE104462). **(C)** *APEX1* correlated with genes in the ferroptosis pathway. (GSE104462). **(D)** The relative mRNA level of APEX1 in HCC38 cells. (GSE154425) (*P < 0.05, n = 3). **(E)** Volcano plot showed significantly differentially expressed genes. (GSE154425). **(F)** *APEX1* correlated with genes in the ferroptosis pathway. (GSE154425).

So how APEX1 regulates the transcription factors? Previous study has reported that the redox function of APEX1 depends primarily on a buried cystine residue. Cys65, Cys93, and Cys99 are necessary for its redox activity, which relates to a redox cycle through the formation of intermolecular disulfide bonds. Among them Cys65 functions as the nucleophilic cysteine, while the others are involved in resolving disulfide bonds that are formed in APEX1 (137). Xanthoudakis et al. have given insight that APEX1 can stimulate AP-1 DNA-binding activity through the conserved Cys residues in Fos and Jun, which may regulate eukaryotic gene expression (138). Cardoso et al. have proven that the binding of signal transducer and activator of transcription 3 (STAT3) to DNA is regulated by the redox function of the APEX1, directly (139). APEX1 inhibition decreased the expression of HIF-1α (93). Nishi et al. have demonstrated that APEX1 can reduce Cys p62 of p50, resulting in the activation of NF-κB DNA binding (140). APEX1 redox function was required for GSK-3β-mediated APEX1 regulation of Nrf2 in Barrett’s related esophageal adenocarcinoma cells (60). Therefore, these transcription factors may be activated by APEX1, presumably through the same redox process. Besides, in order to further illustrate the potential molecular mechanism of APEX1 in ferroptosis, we analyzed transcriptome datasets downloaded from NCBI [GSE104462 (141) and GSE154425 (142)]. It was found that under the action of special ferroptosis inducer erastin, the mRNA level of APEX1 was decreased compared to control group in HepG2 cells (GSE104462) (**Figure 4A**). Also, the mRNA level of APEX1 was decreased compared to control group in HCC38 cells (GSE154425) (**Figure 4D**). Volcano plots showed visualizing expression of different genes screening and cluster analysis (**Figures 4B, E**). Spearman’s correlation of any of two genes was calculated. The genes with a significant correlation (P < 0.05) were found to be the coordinated expression. If two genes resulted in a negative correlation then one gene was concluded to be downregulated, while the other to be upregulated. If two genes resulted in a positive correlation, then they are confirmed either to be down-regulated or up-regulated. **Figure 4C** exhibited that *APEX1* had significant Spearman’s correlations with genes in the ferroptosis pathway, including GPX4, SLC7A11, SLC11A2, voltage dependent anion channel (VDAC2), glutamate-cysteine ligase modifier subunit (GCLM), poly (rC) binding protein 1 (PCBP1), PCBP2, STEAP3 metalloreductase (STEAP3), microtubule associated protein 1 light chain 3 beta 2 (MAP1LC3B2), MAP1LC3C, lysophosphatidylcholine acyltransferase 3 (LPCAT3), glutamate-cysteine ligase catalytic subunit (GCLC), prion protein (PRNP), and acyl-CoA synthetase long chain family member 1(ACSL1) (GSE104462). Also, **Figure 4F** exhibited that *APEX1* had significant Spearman’s correlations with genes in the ferroptosis pathway, including SLC7A11, STEAP3, GCLC, heme oxygenase 1 (HMOX1), ACSL4, and MAP1LC3C. The above data indicated that APEX1 expression decreased significantly in ferroptosis induced by erastin. *APEX1* correlated with genes in the ferroptosis pathway. APEX1 may be involved in ferroptosis, however, the specific mechanisms need further study. Through this review, further understanding of the possible signaling pathways of ferroptosis are undertaken. APEX1 inhibitors or knockdown of APEX1 expression to promote tumor cell death by regulating the ferroptosis pathway can be utilized in the treatment of cancer. Alternatively, we can use APEX1 inducer or overexpression of APEX1 to resist several neurological diseases by inhibiting the ferroptosis pathway. Therefore, we propose that APEX1 may be a potential target for regulating ferroptosis.

Here, we have majorly reviewed the possible regulatory mechanisms of APEX1 in the ferroptosis pathway. So far, there has been no direct evidence that APEX1 can regulate the ferroptosis pathway. The bioinformatics analysis proved that the mRNA level of *APEX1* was decreased in HepG2 cells and HCC38 cells in ferroptosis induced by erastin (**Figures 4A, B**). Through analysis of existing literature, it can be concluded that APEX1 must be involved in ferroptosis through its antioxidant domain. There is an interesting phenomenon: the expression of APEX1 in tumor cells is increased compared to the non-tumor regions, that can resist ROS induced apoptosis. However, the DNA repair function of APEX1 was masked by tumor cells. Therefore, it is worthwhile to explore the role of APEX1 in regulating ferroptosis, especially in the area of cancer. In summary, APEX1 is a multifunctional protein with both important DNA repair and redox capabilities. APEX1 plays key roles in regulation of the ferroptosis pathway, and it is an important potential target for the treatment of various ferroptosis related diseases. We hope that in the future, APEX1 can be used to treat patients and help in improving the condition of the patients suffering from various types of diseases.

## Conclusion

In this review, we highlighted the potential roles of APEX1 in the regulation of ferroptosis. We found that APEX1 plays a vital role in regulating the pathways like system XC-, lipid peroxidation, Nrf2, HIF, and p53. In other words, APEX1 has the potential to regulate cancer, nervous system diseases, and other diseases through ferroptosis. Bioinformatics analysis revealed that the mRNA level of APEX1 was decreased in ferroptosis induced by erastin. *APEX1* correlated with genes in the ferroptosis pathway. Therefore, we have put forward such a panoramic view of APEX1 acting as an inhibitor of ferroptosis.

## Author Contributions

The authors declare no competing financial interests. XL and FZ were responsible for the study concept and design. NG, YZ, YD, and YC drafted the manuscript. XL and FZ provided a critical revision of the manuscript for important intellectual content. All authors contributed to the article and approved the submitted version.

## Funding

This study was financially supported by grants from the Science and Technology Strategic Cooperation Project of the Luzhou People’s Government and Southwest Medical University (No. 2019LZXNYDJ34, 2019LZXNYDJ17, and 2020-JYJ-44) and the undergraduate innovation and entrepreneurship training program (S202110632241). This study was financially supported by school level scientific research project of Southwest Medical University (2021ZKQN008).

## Conflict of Interest

The authors declare that the research was conducted in the absence of any commercial or financial relationships that could be construed as a potential conflict of interest.

## Publisher’s Note

All claims expressed in this article are solely those of the authors and do not necessarily represent those of their affiliated organizations, or those of the publisher, the editors and the reviewers. Any product that may be evaluated in this article, or claim that may be made by its manufacturer, is not guaranteed or endorsed by the publisher.
